# Things Latinas need to plan for safety: A dual-site concept mapping study

**DOI:** 10.1371/journal.pone.0314371

**Published:** 2025-01-10

**Authors:** Alisa Velonis, Maria Jose Baeza, Molly McGown, Daisy Magaña, Jasmine Maldonado, Patricia O’Campo, Nicholas Metheny

**Affiliations:** 1 Division of Community Health Sciences, University of Illinois at Chicago School of Public Health, Chicago, Illinois, United States of America; 2 MAP Centre for Urban Health Solutions, St. Michael’s Hospital Toronto, Toronto, Ontario, Canada; 3 Escuela de Enfermería, Pontificia Universidad Catolica de Chile, Santiago, Chile; 4 University of Michigan Center for Global Health Equity, Ann Arbor, Michigan, United States of America; 5 Access Community Health Network, Chicago, Illinois, United States of America; 6 Rush University Medical Center, United States of America; 7 Dalla Lana School of Public Health, University of Toronto, Canada; 8 Nell Hodgson Woodruff School of Nursing, Emory University, United States of America; COMSATS University Islamabad - Lahore Campus, PAKISTAN

## Abstract

**Background:**

Latina women in the United States experience intimate partner violence (IPV) at high rates, but evidence suggests Latinas seek help for IPV at lower rates than other communities. Safety planning is an approach that provides those experiencing IPV with concrete actions to increase their safety and referrals to formal services. While safety planning is shown to reduce future incidences of violence, little is known about the safety planning priorities of Latinas.

**Approach:**

This study leveraged Group Concept Mapping, a mixed-method process consisting of brainstorming, sorting, rating, and interpreting. First, 17 Latinas who were survivors of IPV and/or professional advocates generated responses to a focal prompt. Next, 19 participants pile-sorted a list of unduplicated responses into categories. Data were analyzed using multidimensional scaling and hierarchical cluster analysis and results were iteratively refined by the study team. Forty-two Latina participants in Chicago and Miami rated each item based on its necessity for safety using two scenarios: if a survivor planned to leave a partner or remain in the relationship. Bivariate correlation analyses were used to examine differences in safety planning priorities across multiple axes. Finally, results were shared with participants for feedback and contextualization.

**Results:**

Combined, a total of 46 Latinas participated in data collection activities. Brainstorming and sorting data generated seven clusters of safety planning needs. Statistically significant differences in cluster rating were found for women who intended to leave a relationship (for whom legal services was most necessary) versus those who intended to stay (for whom safety planning services were critical) and those in Chicago versus those in Miami.

**Conclusions:**

This study contributes to the small, but growing, literature regarding safety planning needs of Latinas in the US and illustrates potential ways in which future safety planning interventions should be tailored to these communities.

## Introduction

Intimate Partner Violence (IPV) constitutes a significant public health problem with far-reaching mental (e.g., depression, substance abuse, suicide risk) and physical (e.g., sexually transmitted diseases, chronic pain, and gastrointestinal disease) health consequences [1–4]. In the United States (US), nearly half of cisgender women (47.3%) in the latest National Intimate Partner and Sexual Violence Survey reported sexual violence, physical violence, and/or stalking by an intimate partner at least once in their lifetime [5]. Too often, IPV is fatal, with more than 3,000 women killed by their partners every year in the US [6, 7].

While Latinas experience IPV at similar rates to other women in the US, research shows that Latinas experience poorer mental and physical health outcomes following IPV compared to other women [4, 8–10]. This is likely due to multiple intersecting structural and interpersonal factors, including strong cultural norms regarding family unity, [11, 12]. greater likelihood of unstable documentation status, [13, 14] overall lower socioeconomic status, [15] and a higher proportion of those with limited English language proficiency. 16 Together, these contribute to Latinas’ hesitancy to share their experiences of violence with informal (e.g., friends, family) and formal (e.g., police, social services, faith leaders) sources and/or seek help for their violent relationships. This cultural norm of “self-silencing” means many Latinas choose to remain in abusive relationships, perpetuating a cycle of violence [16]. Such self-silencing can result in chronic and more severe episodes of violence which, combined with the lack of active screening for IPV by healthcare providers, places Latinas at a disadvantaged position to end a cycle of violence [17]. Given the reasons for which help-seeking for IPV differs in Latinas compared to other groups of cisgender women, strategies aimed at reducing the risk of harm, developing a safety plan while still in the violent relationship, and connecting women to sources of formal and informal support via community or healthcare services are all essential for this population.

To further contextualize the help-seeking needs of US Latinas, this research is situated in two metropolitan areas with large and diverse Latinx populations. Chicago, the country’s third-largest city, contains nearly one million people of Latin American origin, with large communities of Mexican and Puerto Rican immigrants and vibrant ethnic enclaves in the western, northeastern, and southeastern parts of the city [18]. Miami is the country’s largest Latinx-majority metropolitan area, with 69% (~ 1.9 million) of its 2.7 million people identifying as Hispanic or Latinx [19]. Widely seen as a melting pot of Latinx cultures, Miami-Dade County is home to large Caribbean, Central, and South American communities, 54% of whom are foreign-born19. These differences have given rise to varying social services landscapes for people facing IPV, with fewer Latinx-specific resources in Chicago versus Miami. Understanding how the needs of Latinas facing IPV may differ in the context of a majority-immigrant, majority-Latinx community (Miami) compared to one in which Latinas comprise a minority (Chicago) may illuminate how IPV resources can be tailored to specific Latina communities.

Safety planning is a secondary prevention modality that provides those experiencing IPV with concrete actions to increase their safety and referrals to formal legal, social, and health services. Safety planning is one way to empower women to work toward more permanent solutions to IPV [20, 21]. This comprehensive approach encompasses a broad range of strategies aimed at allowing women to take initial steps toward either a) leaving a violent relationship; or b) making a violent relationship safer by connecting women with informal and formal support [22, 23]. The ultimate goal is to empower those experiencing IPV by equipping them with the necessary skills and situational awareness to take control in potentially violent situations [23, 24].

Through safety planning, survivors are provided with guidance on how to best handle challenging situations that commonly lead to violence and reduce the risk of future violence [20, 25]. Notably, its effectiveness depends on considering individual preferences and contextual factors such as the severity of violence and whether the survivor’s life (or those of any children, pets, or others) is at risk [22, 26]. Safety planning can be influenced by a range of significant factors such as whether a person decides to remain in or exit a relationship, [25] the extent of their informal support networks, the nature and duration of the abuse, [23] whether they have children, [25, 26] their legal status, and their capacity to provide financially for themselves and their family [24] For racial and ethnic minority women, integrating culturally specific elements into safety planning interventions are critical to effectively addressing the unique challenges these women face [26].

Despite the availability of online safety planning resources, including one in Spanish, [27]. There remains a significant gap in tailoring these resources to meet the specific needs of Latinas, including their contextual, linguistic, and cultural preferences. To develop effective safety planning interventions for this population, it is crucial to understand Latinas’ safety planning preferences and how these preferences may vary across various aspects of Latina identity. The aim of the present study was to identify and understand the support systems and or resources Latinas experiencing IPV need to be able to plan for their safety, with the goal of developing culturally tailored safety planning resources for the diverse national, racial, and cultural groups that comprise Latina identity.

## Methods

The current study used Group Concept Mapping (GCM) to generate priorities for Latinas’ safety planning needs in Chicago and Miami- two cities with differing and diverse Latinas communities [28]. GCM is a mixed-methods, multi-step process in which participants *brainstorm* responses to a key focal question, then *sort and rate* those items according to a set of pre-defined criteria (such as most important to safety or most likely to be used) [29–31]. Data are analyzed using multidimensional scaling and hierarchical cluster analysis and graphic representations are produced that illustrate the relative importance of each item and the relationships between them. These results are again shared with participants, who help researchers *interpret* and refine the findings.

Initially, the scope of this research was limited to a single city, where participants engaged in the full range of GCM activities following ethics approval from the institutional review board (IRB) of the University of Illinois, Chicago. To explore the ways in which priorities differed across geographic and social service contexts, IRB approval was obtained from the University of Miami and participants from the second city rated the same list of items using the same rating criteria several months later. All recruitment and data collection activities were conducted in Spanish.

### Recruitment

Participants in both cities were recruited through outreach with local community-based organizations that work with Spanish-speaking survivors of partner violence. Flyers with embedded QR codes and/or hyperlinks to an initial, online eligibility screener were created in Spanish and electronically disseminated to organizations with which the researchers have pre-existing relationships with a request that staff share the flyers with current or former clients. Participation was open to survivors of IPV and/or IPV service providers (e.g., shelter employees) who identified as a Latina, were fluent in Spanish (reading and speaking), were over 18, and were able to safely receive communication about the study. Exclusion criteria included being in immediate danger from an abuser. All potential participants were asked to first complete a eligibility screener, which also provided basic study information and collected contact information. Research team members reached out directly to eligible individuals who completed the contact form and/or eligibility screener, and a time was established to review the informed consent via videoconference software or telephone. In Chicago, research team members were also invited to describe the study and pass out flyers with QR codes and hyperlinks directly to participants at community-based organizations that offer services to Spanish-speaking survivors of domestic violence. Given the sensitivity of the content area and out of a concern for safety of the participants, verbal consent was obtained in both cities so that there was no physical evidence of the participant’s participation in the study. Consent was witnessed and documented by the research assistant conducting the rating exercise and a waiver of written informed consent was granted by both IRBs. All recruitment and data collection activities took place between January 6, 2021 and July 13, 2022.

### Demographic survey

All participants were asked to complete a brief demographic survey prior to data collection. Questions included whether they were a survivor of IPV, someone who provides services to survivors of IPV, or both; their age; where they grew up (in or outside of the U.S.); if they live with children under age 18, and their self-identified national identity or ethnicity. These groupings were used to assess potential differences in safety planning needs based on specific axes of identity. Because of the potential sensitivity of these questions for individuals without legal status in the U.S., participants were offered the option of refusing to respond to any question.

### Brainstorming

After participants completed the initial screener and acknowledged consent, they were invited to engage in brainstorming. Using a videoconferencing platform, 17 participants met with research team members individually and responded to the brainstorm prompt *¿Cuándo las personas Latinas están viviendo abuso de pareja*, *cuales cosas necesitarían para planear su seguridad*? (*When Latinas experience abuse by a partner*, *what might they need to plan for safety*?). Seventy-eight individual responses were recorded. At the end of data collection, all responses were deidentified and translated into English. Next, members of the Chicago-based team eliminated duplicate and incomplete or vague responses and grouped items into categories. Working in pairs, the Chicago and Miami-based research teams systematically reduced the remaining list of items by combining similar ideas into a single item and editing for clarity. All changes were reviewed by the entire team, and disagreements were discussed until consensus was reached. This resulted in a final list of 38 unique responses to the focal question. Participants received a $10 gift card in exchange for their time (approximately ten minutes).

### Sorting

After brainstorming activities were completed, the research team prepared for sorting and rating activities by creating a set of sorting and rating instructions in Spanish and scheduling so that a team member was on-hand to walk participants through the online sorting exercise virtually and in-person.

Sorting is used to understand how participants conceptualize the relationships between items. Nineteen women (some of whom participated in the brainstorming exercise) completed the sorting activity, either on-line using Concept System’s Group Wisdom^™^ group concept mapping platform28 or during in-person meetings at one domestic violence advocacy organization. During sorting, each in-person participant was given a set of 38 slips of paper, with one item printed on each piece of paper; they were also given a data collection form on which they would record the final data. Participants were asked to physically sort the 38 items into piles or categories that make sense to them, forming piles with thematically cohesive items and providing a name or description of each category. Once the participants completed the activity and recorded the information onto the data collection form, they turned in the data collection form to a research team member, who later entered it into the Group Wisdom^™^ platform used for concept mapping data analysis. If participants used the Group Wisdom^™^ site directly, they were shown a list of items and asked to sort these into piles on the computer, entering in a description for each pile (instructions were translated into Spanish for all virtual participants, and research team members were available to assist). Participants received a $30 gift card for participating in sorting (approximately one hour).

### Rating

**Fig 1:**
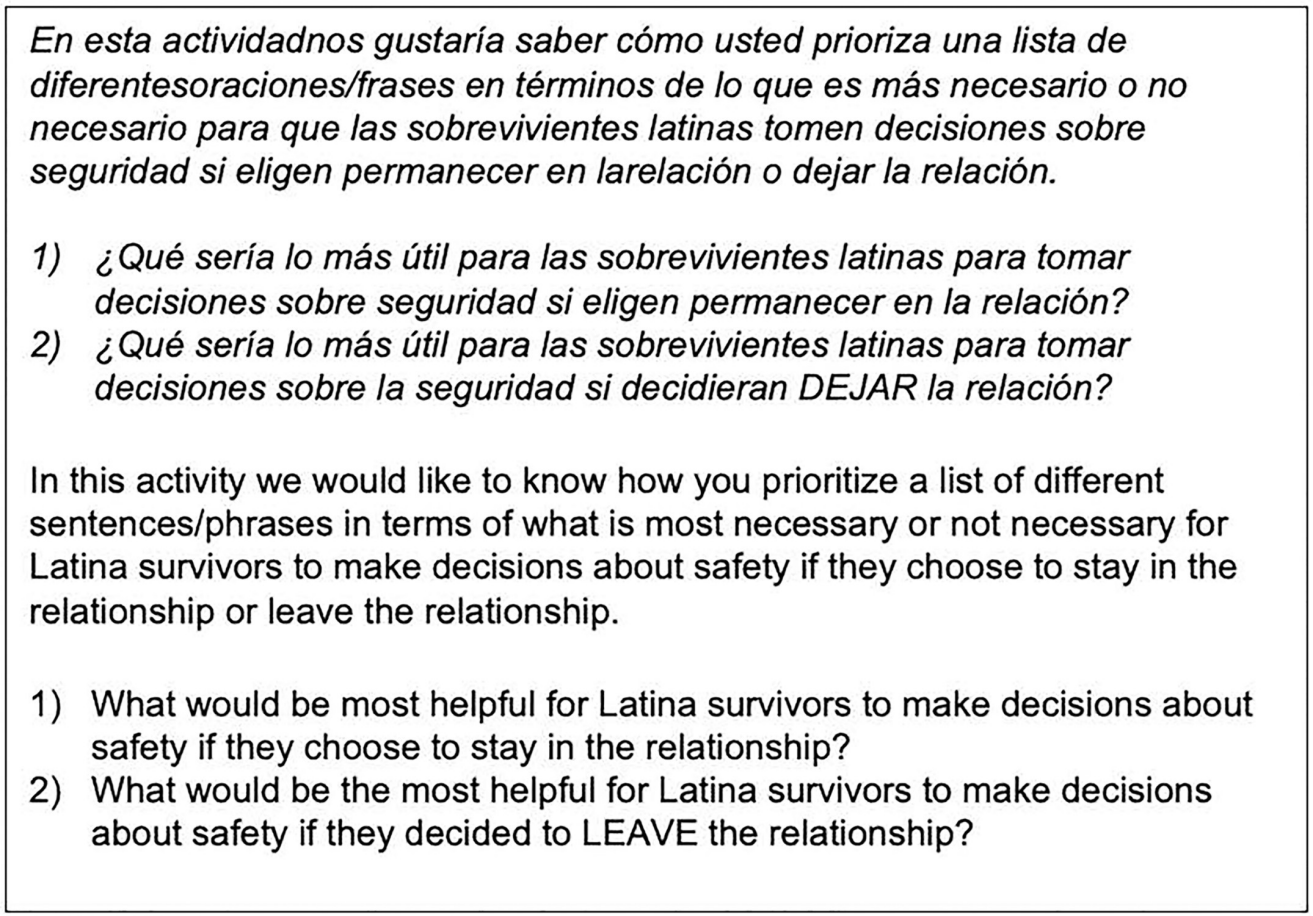
Rating scenarios.

Rating is used to prioritize items using criteria central to the research question. For this study, participants in both Chicago (n = 21) and Miami (n = 21) were provided with a list of items (either virtually, using the Group Wisdom^™^ platform, or physically in person) and asked to rate the importance of each item to a Latina’s ability to make decisions about her safety under two separate scenarios: if the person was planning to leave the abusive partner, and if they were planning to stay with the abusive partner (Fig 1).

Participants were instructed to rate each item on a 5-point Likert-type scale, using as much of the full continuum of the scale as possible (e.g., *all items are important; which items are the most necessary compared to the other items*?). Data from participants who used a single rating score (i.e., who rated every item as a “1” for “most necessary”) were not used in the final analysis, as this was unhelpful with determining the priority of items in relationship to one another (it is also possible that the participants did not understand the instructions). We also reviewed all participants’ responses to determine if patterns of rating responses emerged that suggest that a participant did not think carefully about their responses (e.g., a participant answered question 1 with a “1”, question 2 with a “2”, etc.). Participants were offered $10 gift card for completing all rating activities. The total time needed to complete all rating activities was approximately 30 minutes.

### Analysis

**Fig 2:**
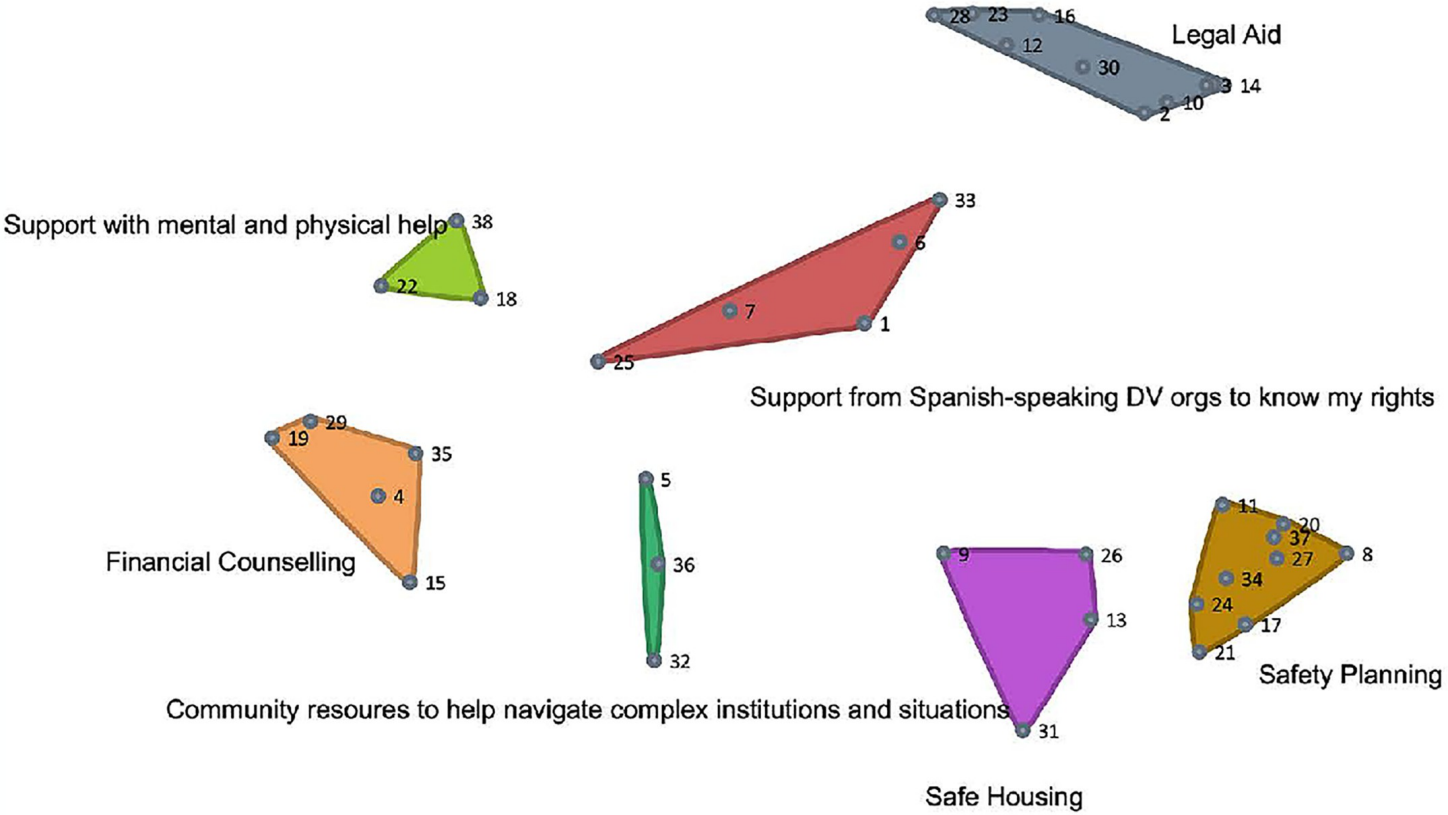
Final cluster map (each number represents an individual item).

Rating data from Miami were de-identified and securely transferred to the Chicago team for analysis. Sorting data from Chicago and rating data from both sites were analyzed using the Group Wisdom^™^ concept mapping platform. First, multidimensional scaling was applied to the sorting data, producing a visual “point map” that spatially illustrates the relatedness of each item to other items (these are the individual dots in Fig 2). Next, hierarchal cluster analysis was used to generate thematic categories based on the frequency of items being sorted together. These categories are represented as “clusters” of items on the point map (Fig 2). In-depth descriptions of multidimensional scaling and cluster analysis—including their use in concept mapping—have been described in detail elsewhere [32–35].

GCM’s cluster analysis stage provides multiple potential ways in which the sorting data may be grouped, with each iteration of analysis merging two clusters into one. As a truly mixed method approach, it is then up to the research team to review each of these maps and decide, based on expert knowledge and community consultation, which model best represents the phenomenon being studied [32, 35]. First, we determined that a similarity cutoff of 1 (meaning that individual items must have been sorted together by at least two participants to be considered to have a relationship) enhanced the ability to interpret the sorting data and provided greater confidence in the relationship between two items (stress value = 0.2218). Next, the Chicago and Miami research teams reviewed ten iterations of cluster analyses, beginning with a map of 14 clusters and ending with a map of four clusters (with each analysis reducing the number of clusters by one). By beginning with a large number of clusters (in which some items are left alone or at most paired with another item) and combining two sets of clusters together with each iteration, researchers can clearly see the formation of themes based on how participants sorted the items. We began by having each individual team member reviewing what changed when 14 clusters were reduced to 13, when 13 were reduced to 12, etc. As a larger group, we then discussed each iteration, reaching consensus that a seven-cluster solution represented the best fit for the data, based on the intention of the research and the thematic cohesion of items within each cluster. Each cluster was assigned a number, as well as a description of the construct it represented (e.g., Cluster 1: “Support from Spanish-Speaking Organizations to Know My Rights”).

Next, bivariate correlation analysis using the rating data allowed us to examine differences in priorities across multiple axes. Specifically, we explored how priorities changed if the survivor in the scenario was planning to stay in or leave the relationship, and we stratified this analysis based on city (Chicago or Miami). T-tests were conducted to identify statistically significant cluster rating differences.

In addition to using clusters as our unit of analysis, we also explored the prioritization of individual items. GCM allows us to create bivariate scatter plots (called “go-zones” in GCM parlance [29]) correlating the mean scores of individual items across two constructs (e.g., necessity when staying vs. necessity when leaving, or Chicago participants vs. Miami participants). By dividing the plot graph into four quadrants based on the overall mean rating score for each construct, we can see which of the individual items are considered “most necessary” and “least necessary” for one or both of those constructs (scatter plots not show). Those items that fall within the quadrant of “most necessary” for both constructs are considered within the “go-zone” and those are likely to be the specific items that will be important to include in our electronic safety plans, guiding us in efforts to tailor safety plans across axes of identity.

### Interpretation/member checking

In Chicago, a group of five Latinas who had participated in at least one previous GCM activity met virtually with the research team. The group reviewed the purpose of the research and the list of 38 items. Next, the research team shared the point map with participants, explaining that items that were physically close together had been sorted together more often than items that were far apart. Finally, the group looked in detail at the individual clusters, discussing whether the items belonged together, why, and generating a name for the cluster.

### Findings

Participant demographics are shown in Table 1. Combined, a total of 46 Latinas participated in one or more data collection activities. Most participants from Chicago identified as Mexican or Mexican American, most grew up in a country outside of the U.S., and most were over 35 years of age. Most Miami participants chose to write in their country of origin or not to report this information. They were also slightly older, on average, than the Chicago participants.

**Table 1:** Participant demographics.

	Chicago: N = 25 total			Miami
	Brainstorming*	Sorting**	Rating**	Rating only
Total	n = 17	n = 19	n = 21	n = 21
Provider Only	8	5	5	-
Survivor Only	7	11	12	20
Provider and Survivor	2	1	2	-
Preferred to not Respond	-	2	-	1
Missing	-	-	2	-
**Age:**				
18–24	1	1	1	-
25–34	6	3	3	2
35–44	5	7	8	7
45–54	1	2	2	6
55 +	4	4	5	6
Preferred to not Respond		2	-	-
Missing	-	-	2	
**Participant grew up…**				
In or mostly in the US	9	5	7	1
In or mostly in another country	8	12	12	14
Preferred to not Respond		2	-	-
Missing	1	-	2	6

* Twenty-two eligible individuals completed the online screener during the brainstorming phase.

** Twenty-nine eligible individuals were assigned to Sorting and/or Rating activities in Group Wisdom.

The final list of items is displayed in Table 2, and the final cluster map is displayed in Fig 2. Items were clustered into seven categories representing: *legal aid*, *safety planning*, *safe housing*, *financial counseling*, *mental and physical health assistance*, *support from Spanish-speaking organizations to help understand rights*, and *community resources that would help navigate complex institutions and situations*. The average rating score for each cluster is provided for each of the rating scenarios (staying vs. leaving), and scores are also stratified by population. Finally, the average rating score for each individual item is also provided.

**Table 2:** Concept mapping statements and average rating scores, by cluster.

Item #	Statements	Rating Score Average					
		Combined		Chicago		Miami	
		Stay	Lv	Stay	Lv	Stay	Lv
	**Cluster 1—Support from Spanish Speaking Organizations to Know My Rights**	1.86	1.96	1.86	2.01	1.85	1.85
1	**Necesitan una organización que los oriente o guie sobre resolver los conflictos familiares.** They need an organization to guide or guide them in resolving family conflicts.	1.91	2.03	1.75	2.16	2.17	1.80
6	**Necesitan conocer las leyes de vivienda que pueden ayudar a las sobrevivientes de violencia domestica (por ejemplo, cuando romper un contrato de arrendamiento, qué hacer cuando ha sido discriminado debido a la violencia doméstica, etc.).** They need to know the housing laws that can help people survivors of domestic violence (for example, when breaking a contract leasing, what to do when you have been discriminated against due to violence domestic, etc.).	2.03	2.04	2.30	2.00	1.58	2.11
7	**Necesitan acceso a consejeros de habla hispana de organizaciones de violencia doméstica o abuso sexual que puedan ofrecerles apoyo y recursos.** They need access to Spanish-speaking counselors from domestic violence or sexual abuse organizations that may offer you support and resources.	1.53	1.76	1.45	1.84	1.67	1.60
25	**Necesitan a alguien que hable español que pueda ayudarlos a navegar por instituciones complejas (por decir, bancos, compañías telefónicas, agencias gubernamentales)** They need someone who speaks Spanish who can help them navigate complex institutions (i.e. banks, phone companies, government agencies)	2.09	2.07	2.10	2.05	2.08	2.10
33	**Necesitan información sobre las leyes estatales que protegen a los sobrevivientes que necesitan ausentarse del trabajo para manejar problemas relacionados con la violencia doméstica.** They need information on state laws that protect survivors who need to take time off work to handle problems related to domestic violence.	1.72	1.89	1.70	2.00	1.75	1.63
	**Cluster 2—Support with Mental and Physical Health**	1.94	1.82	1.93	**2.04**	1.93	**1.40**
18	**Necesitan acceso a la terapia de un consejero que hable español y que tenga experiencia trabajando con mujeres y niños que han vivido violencia de pareja.** They need access to therapy from a counselor who speaks Spanish and has experience working with women and children who have experienced intimate partner violence.	1.72	1.69	1.60	1.79	1.92	1.50
22	**Necesitan acceso a atención médica en español.** They need access to medical care in Spanish.	2.19	1.86	2.15	2.26	2.25	1.10
38	**Necesitan la ayuda de alguien que hable español que pueda ayudarlos a obtener los servicios médicos que necesiten.** They need the help of someone who speaks Spanish who can help them get the medical services they need.	1.9	1.9	2.05	2.05	1.64	1.60
	**Custer #3—Financial Counseling**	2.04	1.90	2.03	1.95	2.05	1.82
4	**Necesitan ayuda económica de emergencia para poder tomar decisiones que no dependan del apoyo de su pareja.** They need emergency financial help to be able to make decisions that do not depend on the support of their partner.	1.72	1.59	1.60	1.58	1.92	1.60
15	**Necesitan información sobre cómo abrir una cuenta bancaria separada y otras formas de ahorrar y administrar fondos de manera discreta.** They need information on how to open a separate bank account and other ways to save and manage funds discreetly.	2.06	2.03	2.00	2.32	2.17	1.50
19	**Necesitan ayuda para conseguir que un empleador les dé tiempo libre o horario flexible para ocuparse de los problemas relacionados con la violencia doméstica.** They need help getting an employer to give them time off or flexible hours to deal with issues related to domestic violence.	2.23	2.14	2.47	2.16	1.83	2.10
29	**Necesitan ayuda para conseguir un trabajo si no lo tienen** They need help getting a job if they don’t have one	2.13	1.86	2.05	1.89	2.25	1.80
35	**Necesitan tener acceso a transporte para poder ir y venir de los servicios y las citas.** They need access to transportation to get to and from services and appointments.	2.06	1.9	2.05	1.79	2.09	2.10
	**Cluster 4—Community resources to help navigate complex institutions and situations**	2.18	2.10	2.15	2.19	2.22	1.93
5	**Necesitan opciones de cuidado de niños con las que se sientan cómodas.** They need childcare options that they are comfortable with.	2.0	1.86	2.10	1.95	1.83	1.70
32	**Necesitan saber cómo obtener documentos importantes que aún no tienen.** They need to know how to get important documents that they don’t have yet.	2.06	1.97	1.9	2.11	2.33	1.70
36	**Necesitan a alguien que pueda ayudarles a revelar / explicar su situación a sus familiares y amigos** They need someone who can help them reveal / explain their situation to their family and friends	2.47	2.48	2.45	2.53	2.50	2.40
	**Cluster 5—Legal Aid**	1.99	1.73	1.97	1.69	2.04	1.80
2	**Necesitan que todos los procedimientos judiciales (corte) sean completamente accesibles en español (como tener un intérprete y / o las indicaciones automáticas, si la corte se lleva a cabo virtualmente).** They need all judicial (court) proceedings to be fully accessible in Spanish (such as having an interpreter and / or automated directions, if the court is held virtually).	2.00	1.45	1.95	1.58	2.08	1.20
3	**Necesitan información en español sobre sus derechos con respecto a la custodia de los hijos.** They need information in Spanish about their rights regarding child custody.	1.72	1.55	1.65	1.53	1.83	1.60
10	**Necesitan saber cómo los problemas legales (por ejemplo, órdenes de protección, casos penales, divorcio, custodia compartida, derechos de visita, reclamos de custodia, etc.) pueden afectar el estado migratorio.** They need to know how legal problems (for example, warrants, protection, criminal cases, divorce, joint custody, visitation rights, custody	1.94	1.93	1.75	1.79	2.25	2.25
12	**Necesitan a alguien que hable español que pueda asistirlos obtener una orden de protección (por ejemplo, alguien que pueda ayudarlos a completar formularios o ir con ellos a la corte).** They need someone who speaks Spanish who can assist them in obtaining an order of protection (for example, someone who can help them fill out forms or go to court with them).	1.94	1.90	1.90	1.84	2.00	2.00
14	**Necesitan información en español sobre separación y divorcio.** They need information in Spanish about separation and divorce	2.13	1.97	2.05	1.95	2.25	2.00
16	**Necesitan defensores legales que hablen español para ayudarlos con asuntos legales que no requieren a un abogado.** They need legal advocates who speak Spanish to help them with legal matters that do not require an attorney.	1.88	1.93	1.95	1.95	1.75	1.90
23	**Necesitan acceso a abogados bilingües especialistas en violencia domestica que puedan ayudarlos con asuntos de derecho civil, como el divorcio o la custodia de los hijos.** They need access to bilingual domestic violence attorneys who can help them with civil law matters, such as divorce or child custody.	2.13	1.52	2.10	1.44	2.17	1.67
28	**Necesitan un abogado de inmigración que hable español que pueda ayudarlos a navegar las opciones legales relacionadas con problemas de inmigración (por ejemplo, ayuda para obtener una VISA U) a bajo costo o sin costo alguno.** They need a Spanish-speaking immigration attorney who can help them navigate legal options related to immigration issues (for example, help obtaining a U-VISA) at little or no cost.	2.13	1.86	2.20	1.84	2.00	1.90
30	**Necesitan información en español sobre dónde y cómo obtener una orden de protección (incluyendo un reporte de policía si es necesario).** They need information in Spanish about where and how to get a warrant for protection (including a police report if necessary).	2.09	1.45	2.15	1.32	2.00	1.70
	**Cluster 6—Safety Planning**	1.72	1.95	**1.50**	2.02	**2.10**	1.81
8	**Necesitan hablar con los niños sobre qué hacer en caso de emergencia (cuándo llamar al 911 o un conocido cercano)** They need to talk to children about what to do in an emergency (when to call 911 or a close acquaintance)	1.72	2.00	1.40	1.89	2.25	2.20
11	**Necesitan un plan de seguridad personalizado que esté en español que describa las acciones que pueden tomar para mantenerse a salvo si se quedan con su pareja y / o si dejan a su pareja** They need a personalized safety plan that is in Spanish that describes the actions they can take to stay safe if they stay with their partner and / or if they leave their partner	1.59	2.00	1.40	1.95	1.92	2.10
17	**Necesitan tener documentos importantes guardado en algún lugar al que puedan acceder fácilmente (donde el abusador no se dará cuenta o no podrá acceder)** They need to have important documents stored somewhere that they can easily access (where the abuser will not notice or cannot access)	1.56	1.76	1.35	1.74	1.92	1.80
20	**Necesitan saber las acciones específicas que pueden tomar para mantenerse a salvo mientras viven en la casa con una pareja abusiva (por ejemplo, habitaciones en las que no quieren estar si su pareja está enojada).** They need to know the specific actions they can take to stay safe while living in the house with an abusive partner (for example, rooms they don’t want to be in if their partner is angry).	1.75	2.14	1.60	2.47	2.00	1.50
21	**Necesitan tener una bolsa de emergencia empacada y escondida o ubicada con un amigo/familiar con cosas esenciales para ellos y sus hijos en caso de que tengan que salir de casa rápidamente (por ejemplo, medicamentos, ropa, pañales, etc.).** They need to have an emergency bag packed and hidden or located with a friend / family member with essentials for them and their children in case they have to leave home quickly (eg, medications, clothing, diapers, etc.).	1.61	1.68	1.40	1.74	2.00	1.56
24	**Necesitan una lista de contraseñas e información de datos personales importante (números de teléfono, códigos de acceso a edificios, etc.).** They need a list of passwords and important personal data information (phone numbers, building access codes, etc.).	1.97	2.10	1.70	2.32	2.42	1.70
27	**Necesitan poder reconocer los señales de peligro de sus parejas y cuando es probable que su pareja reaccione con violencia.** They need to be able to recognize the danger signs of their partners and when your partner is likely to react violently.	1.71	2.00	1.35	2.11	2.36	1.80
34	**Necesitan una aplicación en el teléfono para alerta a alguien que puede ayudar cuando uno está en peligro.** They need an app on the phone to alert someone who can help when one is in danger.	1.84	2.07	1.75	2.26	2.00	1.70
37	**Necesitan saber qué documentos importantes deberán llevar consigo si tienen que salir de casa rápidamente.** They need to know what important documents to take with them if they have to leave home quickly.	1.71	1.79	1.55	1.74	2.00	1.90
	**Cluster 7—Safe Housing**	2.00	1.71	2.04	1.71	1.93	1.71
9	**Necesitan recursos para las sobrevivientes de violencia doméstica que necesitan un lugar para quedarse (por ejemplo, refugios locales, cómo obtener fondos para la vivienda, materiales para empacar, compañías de mudanzas).** They need resources for survivors of domestic violence who need a place to stay (eg, local shelters, how to obtain housing funds, packing materials, moving companies).	2.09	1.48	2.05	1.32	2.17	1.80
13	**Necesitan amigos o familiares que puedan proveer un lugar para quedarse temporalmente.** They need friends or family who can provide a place to stay temporarily.	2.06	2.07	2.20	2.26	1.83	1.67
26	**Necesitan información sobre diferentes refugios u otras opciones de vivienda y sus restricciones (por ejemplo, si tienen personal que habla español o acepta niños varones o pueden adaptarse a necesidades médicas).** They need information about different shelters or other housing options and their restrictions (for example, if they have staff who speak Spanish or accept children male or can be adapted to medical needs).	1.94	1.52	1.95	1.47	1.92	1.60
31	**Necesitan tener una tarjeta de crédito o débito, efectivo y / o algunos objetos de valor en caso de que necesiten irse rápidamente.** They need to have a credit or debit card, cash, and / or some valuables in case they need to leave quickly	1.90	1.79	1.95	1.79	1.82	1.78

Boxes around cluster averages indicate significant difference between Staying/Qudar and Leaving/Dejar (p<0.05).

Bolded cluster averages indicate significant difference between Chicago and Miami (p<0.05).

### Cluster findings

#### Quedar versus Dejar (Staying versus leaving)

**Fig 3:**
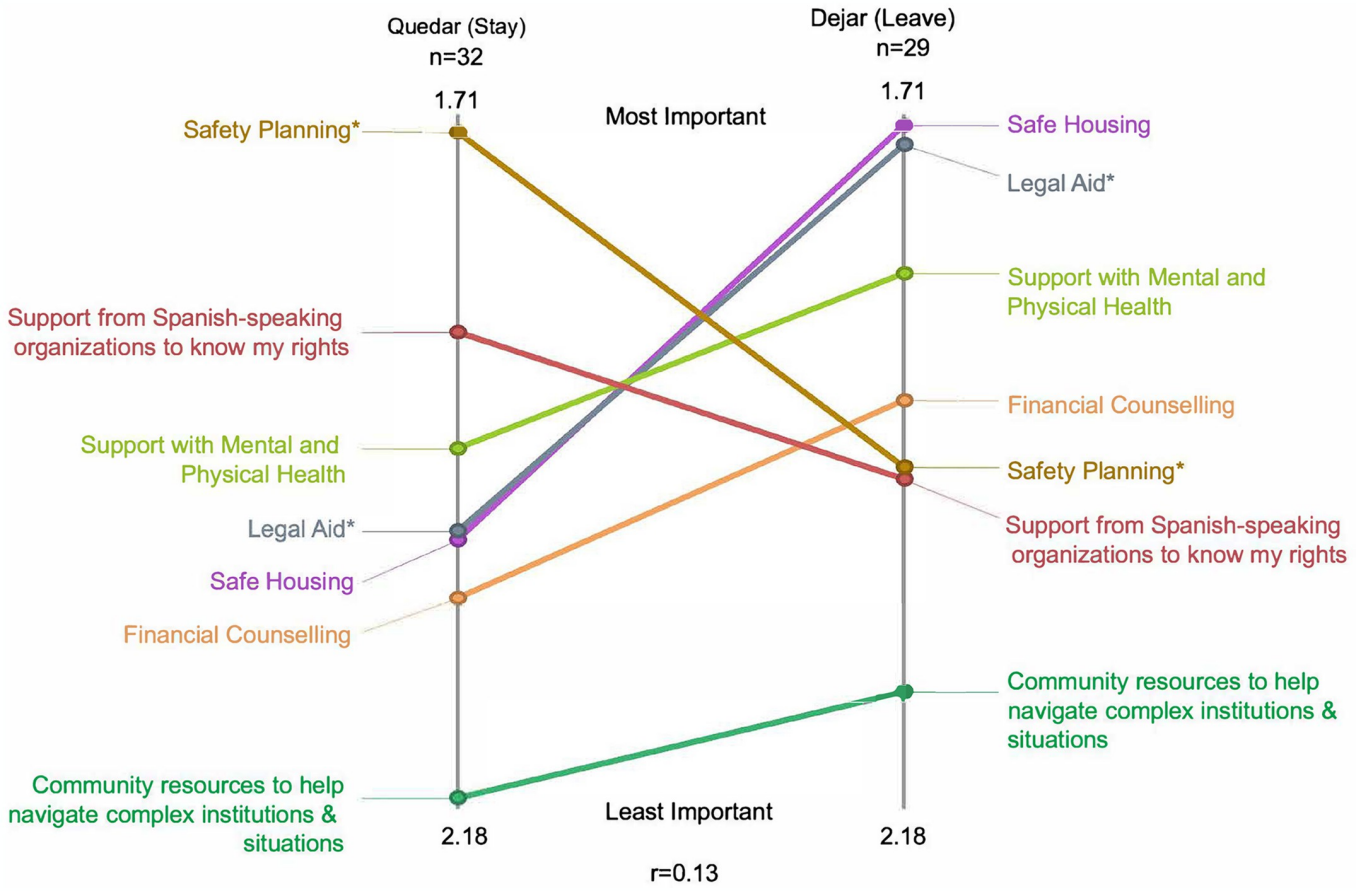
Pattern match illustration of cluster ratings comparing quedar (stay) and Dejar (leave) scenarios, Chicago and Miami participants.

Overall, we found a very weak correlation between the types of supports Latina survivors would need to plan for safety if they intended to leave a partner compared to if they intended to remain with a partner (r = 0.13). Fig 3 illustrates these findings using a “pattern match”, or a ladder graph representation of the data. When survivors were intending to stay in a relationship, *safety planning* was considered the most important (necessary) priority (average rating score 1.71). When survivors were planning to leave a relationship, *legal aid* was ranked most important (average ratings scores 1.71 and 1.73, respectively). The safety planning (t = 3.52, p<0.005) and legal aid (t = 3.16, p<0.01) clusters significantly different when respondents were asked to respond as if they were staying or leaving the abusive relationship. The statistical significance of this finding remained when Chicago and Miami participants were analyzed separately (not shown). Additionally, Miami participants rated *support with mental and physical health* and *safe housing* as significantly more necessary for Latina survivors planning to leave a relationship (compared to staying), which was not statistically significant among Chicago participants.

#### Chicago versus Miami

When considering the scenario of Latina survivors who intend to stay in their relationship, we again found very weak correlation (r = 0.12) between Chicago and Miami. One notable exception was of the importance of *safety planning*, which Chicago participants rated as significantly more necessary (1.50) compared to Miami participants (2.10) (t = 7.67, p<0.001). Likewise, we found very weak correlation (r = 0.11) between Chicago and Miami when it came to considering the priorities of Latina survivors planning to leave a relationship. Again, only one cluster differed significantly in priority. Miami participants rated *support with mental and physical health* as significantly more important when leaving (1.40) than Chicago participants.

**Fig 4:**
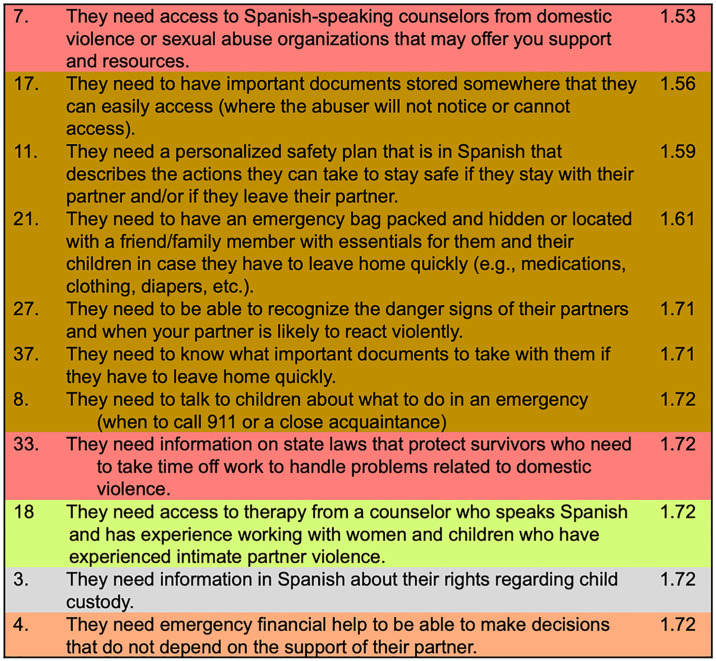
Most important items when survivors are *planning to stay in an abusive relationship*, in rank order of importance, Chicago and Miami participants.

*Individual item findings*. For survivors who are planning to stay in their relationship, we identified 11 individual items that fell above the mean necessity score (i.e., that fell within the “go zone”) for participants in both Chicago and Miami. Six of these items were from the *safety planning* cluster, two were from the *support from Spanish-speaking organizations to know my rights* cluster, and one each from the *legal aid*, *support with mental and physical health*, and *financial counselling* clusters (Fig 4).

**Fig 5:**
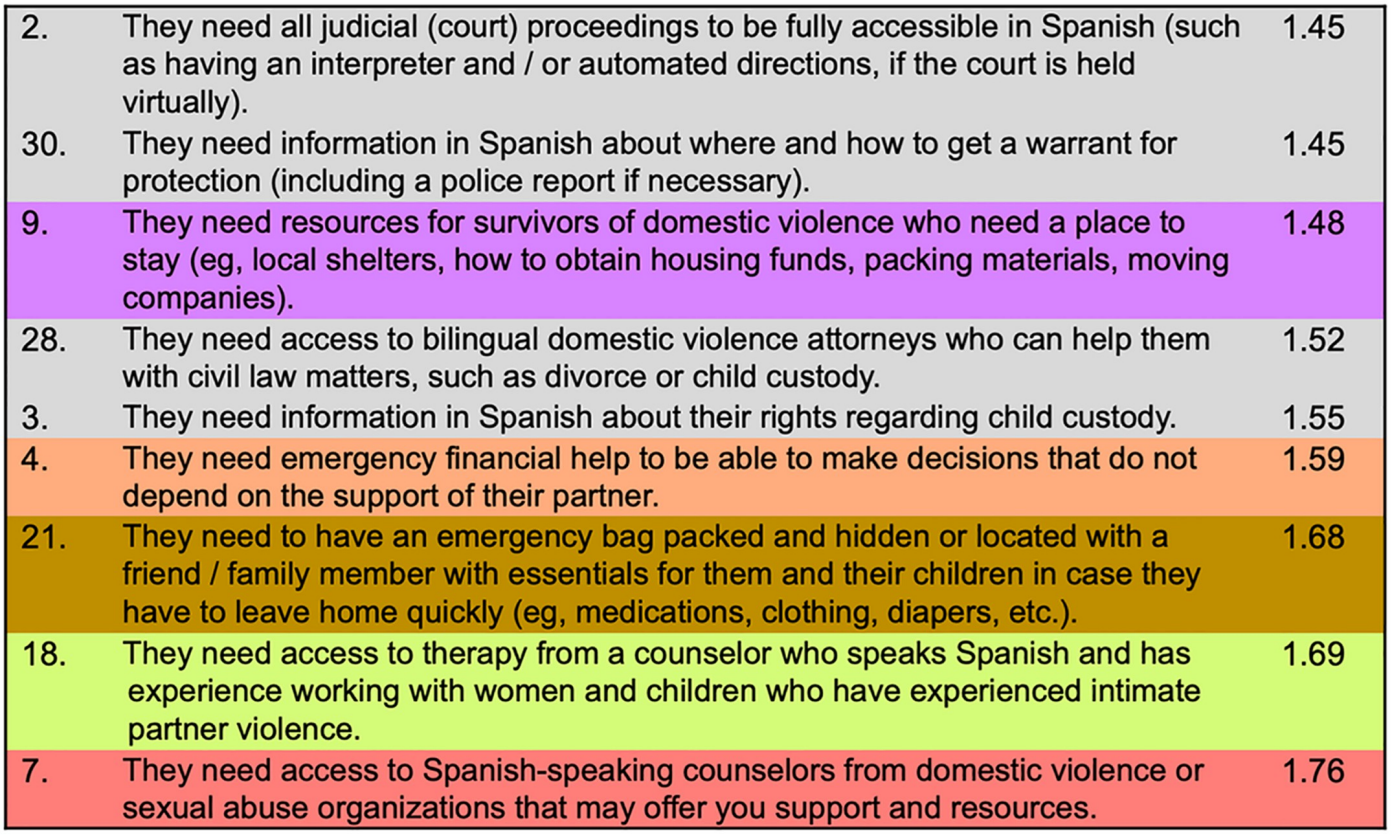
Most important items when survivors are *planning to leave an abusive relationship*, in rank order of importance, Chicago and Miami participants.

For survivors who are planning to leave the relationship, nine individual items fell above the mean necessity score (i.e., the “go zone”) for participants in both Chicago and Miami. Four of these items were from the *legal aid cluster*, and one each from the *support from Spanish-speaking organizations to know my rights*, *support with mental and physical health*, *financial counselling*, *safety planning*, and *safe housing clusters* (Fig 5).

## Discussion

This study represents some of the first data on the specific IPV help-seeking needs of Latinas residing in the US. Participants initially proposed 78 responses to the brainstorming question (38 of which were considered unique), indicating both broad and high levels of safety planning needs. Sorting and rating participants revealed additional insights regarding how safety planning needs may vary based on two important axes: desire to stay in or leave the relationship and city of residence.

Regarding the desire to stay in or leave a violent relationship, significant differences were noted between the decision to stay or leave the relationship. *Safety planning* was considered the most important priority when survivors were planning to stay, while *legal aid* was ranked most important when survivors were planning to leave a relationship. There are likely several intersecting cultural and practical factors that alter safety planning needs in Latinas on this axis. For Latinas who desire to stay in a relationship despite its violent nature, theoretical and empirical literature suggest cultural forces such as *Marianismo* and *Familismo* that prize selflessness, humility, and a strong dedication to the family unit may supersede women’s individual desires to exit a violent relationship [11, 12]. Also, women may truly desire for the violent partner to change the behaviors (e.g., substance use) that accompany the violence, rather than ending the relationship itself and remain committed to helping the violent partner through this process [36]. Latina women may also be economically dependent on the violent partner, making safety planning resources that promote financial independence via opening independent bank accounts, earning money independently, and accessing joint resources independently especially important for those who do not desire to stay, but feel unable to leave for financial reasons [37]. Conversely, past empirical evidence shows that the need for *legal aid* arises when Latina women are contemplating the possibility of leaving the relationship, and when having concerns about child safety, factor that has been recognized as a significant driver for leaving the relationship [38, 39].

Significant differences were also noted by geography, despite no statistically significant differences in age, education, or immigration status of the participants in Chicago and Miami. Participants rated several priorities differently by city of residence, with two priorities rising to statistical significance. Participants in Chicago rated the *safety planning* cluster (comprised of statements on practical actions to take when violence arises) significantly higher than those in Miami, while Miami-based respondents rated *support with mental and physical health* (comprised of statements on accessible Spanish-language healthcare resources) as significantly more important than those in Chicago. While the reasons for these differences are likely complex, one reason for these findings could be the differing social services landscapes of both cities. Evidence suggests that Latinas in Chicago and other northern cities with substantial, but minority, Latinx communities have high levels of social isolation- especially for immigrant Latinas- and fewer social services that cater to the specific needs of this community [40]. This could lead to a stated importance on safety planning behaviors that require individual, self-directed actions in Chicago versus in majority-Hispanic Miami where social networks may be more substantial. Conversely, Miami-based participants who rated the need for mental and physical health services in Spanish may view this as more important given the preponderance of Spanish-speaking healthcare providers and Latinx-focused healthcare services in South Florida versus Chicago [41]. Conversely, this finding may reflect differing priorities of Latina-serving service providers versus community members. Since Chicago participants included both community members and IPV service providers, these results may be skewed by the opinions of providers who often interact with women who need immediate, individualized safety planning actions and are at the point of more severe violence.

Despite these distinctions, there were more similarities in responses than differences. Most clusters were not significantly different by geography or desire to leave, and individual item analysis revealed overlapping priorities in two key areas: legal aid and physical and mental health. Regardless of whether they desire to leave the relationship or not, Latinas should have easy, affordable access to legal support that is agnostic to the legality of their immigration status. Spanish-language legal aid for those living with IPV is commonly cited as lacking among diverse groups of Latinas in the US and should be built into safety planning interventions for this community [39, 42, 43]. Despite rating support for physical and mental health differently compared to other priorities, women in Chicago and Miami agreed it was an important component of safety planning overall. Resources that prioritize personalized handoffs to trusted, locally based, Spanish-speaking mental and physical health providers may improve the utility of safety planning interventions for Latinas.

### Limitations

There are several limitations to note. First, a surge in COVID-19 cases at the time of data collection precluded additional in-person data collection in Chicago and severely limited it in Miami. This led to a smaller-than-expected sample size and limited the sample to those with access to virtual means of participation. Second, Miami-based participants did not engage in the first (brainstorming) and second (sorting) step of GCM. While the prioritization steps of sorting and rating are the most critical for our purposes, additional responses and insights could have been gleaned from these participants. Importantly, this is a common approach in Concept Mapping studies and should not be seen to invalidate the overall findings. Third, participants used a small range when rating the importance of the clusters. Despite instructions to use the entirety of the rating scale, participants rated each cluster high on the scale, indicating a large need for additional safety planning services, but limiting the utility of the rating exercise and in some cases resulting in data not being included (such as when a participant rated every item as equal in importance). While this is not uncommon in GCM studies, it should be noted as a limitation. Future GCM efforts with this community should be more explicit about using the full rating continuum. Finally, there are additional factors related to participants’ identity that remain unaccounted for. These include the recency and circumstances around immigrant respondents’ migration and documentation status. The latter was advised by community members to be omitted, given sensitivities around documentation status, but could be an additional axis on which safety planning needs differ.

### Strengths

Strengths of this study include its use of GCM methods and diverse sample of Latinas in two major US cities. GCM is a robust methodology for identifying and prioritizing the safety needs and decisional considerations of women, and it was easily adapted to virtual methods of data collection when physical distancing measures were in place. This methodology safely allowed for virtually identical data collection using in- person and virtual engagement with diverse participants who had varied life experiences and histories of IPV. This work constitutes some of the first GCM data among Latina IPV survivors and allowed for an iterative conceptualization of how Latinas understand and prioritize their safety needs within their lived experiences. Its “democratic” nature ensured all participants had an equal voice in the brainstorming, rating, and sorting processes, and member checking allowed for sound interpretation of the GCM results in concert with community members. Including Latinas from two cities with differing and diverse Latinx communities, as well as Latina-serving IPV service providers, allowed for additional insights and generalizability.

## Conclusions

Although several differences are highlighted, safety planning needs are largely congruent among this sample of Latina IPV survivors and IPV service providers in Chicago and Miami, though significant differences exist depending on whether survivors are planning to stay or leave a violent relationship. This finding suggests that safety planning apps should take survivors’ relationship intentions into account. While our research found that plans may want to prioritize safety planning needs (such as keeping documents and resources packed, having a personalized plan in Spanish, and access to advocates who could help them) when survivors intend to remain with a violent partner, and prioritize Spanish-language legal assistance (including help with court proceedings, protection orders, and child custody) and housing for survivors who plan to leave a violent relationship, the reluctance of participants to consider any item *not necessary* clearly suggests that safety plans for Latinas should include the full range of safety information.

This study contributes to the small, but growing, literature regarding safety planning needs in marginalized communities of women in the US. Culturally and linguistically tailored safety planning resources for Latinas remain scarce and additional resources should be developed in concert with Latina communities to ensure they reflect the priorities and needs of specific, local groups. These resources could draw on a universal template that broadly addresses safety planning in Latina communities while adding locally tailored modules. Ideally, apps and other resources would provide referrals to established organizations that Latinas trust and feel safe accessing. While preliminary, these results are a steppingstone to understanding how to best tailor such resources to this marginalized and under-resourced community.
